# Successful Treatment of Central Nervous System Lymphoma with Combination Therapy of Nimustine and Prednisolone in Two Dogs

**DOI:** 10.3390/vetsci10090533

**Published:** 2023-08-22

**Authors:** Yuko Mizutani, Yoshiyuki Inoue, Yoshimichi Goda, Shinya Mizutani, Taketoshi Asanuma, Naoki Miura, Yuichi Hidaka, Reiichiro Sato, Hiroyuki Satoh

**Affiliations:** 1 Faculty of Agriculture, Veterinary Teaching Hospital, University of Miyazaki, 1-1 Gakuen Kibanadai-nishi, Miyazaki-shi 889-2192, Miyazaki, Japan; 2 Faculty of Agriculture, University of Miyazaki, 1-1 Gakuen Kibanadai-nishi, Miyazaki-shi 889-2192, Miyazaki, Japan; 3 Graduate School of Medicine and Veterinary Medicine, University of Miyazaki, 1-1 Gakuen Kibanadai-nishi, Miyazaki-shi 889-2192, Miyazaki, Japan; 4 Faculty of Veterinary Medicine, Okayama University of Science, 1-3 Ikoinooka, Imabari 794-8555, Ehime, Japan; 5 Joint Faculty of Veterinary Medicine, Veterinary Teaching Hospital, Kagoshima University, 1-21-24 Korimoto, Kagoshima 890-0065, Kagoshima, Japan

**Keywords:** canine, chemotherapy, intracranial, lymphoma, nimusutine

## Abstract

**Simple Summary:**

Of intracranial tumors, primary central nervous system lymphoma (PCNSL) rarely occurs in dogs. Here, we describe two dogs with neurological symptoms and intracranial disseminated lesions that appeared on magnetic resonance imaging (MRI). PCNSL or secondary central nervous system lymphoma was diagnosed on the basis of MRI findings and cerebrospinal fluid examinations. Nimustine (ACNU) is a nitrosourea alkylating agent, a class of drugs that includes lomustine. The dogs in this study were treated with combined chemotherapy comprising nimustine and prednisolone, which achieved complete or nearly complete remission of neurological symptoms and long-term survival (>2583 days and 1218 days), but with some problematic adverse effects.

**Abstract:**

Of intracranial tumors, primary central nervous system lymphoma (PCNSL) is rare in dogs. Herein, we describe our experience with two dogs (a 3-year-old intact female toy poodle and a 5-year-old spayed female toy poodle) that developed neurological symptoms. Magnetic resonance imaging (MRI) revealed intracranial disseminated lesions. Cerebrospinal fluid (CSF) examination revealed pleocytosis and B-cell monoclonal proliferation in both cases. PCNSL or secondary central nervous system lymphoma (SCNSL) was diagnosed on the basis of MRI findings and CSF examinations. Nimustine (ACNU) is a nitrosourea alkylating agent, a class of drugs that includes lomustine. Nimustine is mainly used to treat human intracranial neoplasia because of its high permeability across the blood-brain barrier. The dogs in this study were treated with combined chemotherapy comprising nimustine and prednisolone, which achieved complete or nearly complete remission of neurological symptoms and long-term survival (>2583 days and 1218 days), but with problematic adverse effects. We determined that the dose of nimustine for canine PCNSL or SCNSL with intravenous infusion was 25–30 mg/m^2^ every 3–4 weeks for a total of four times; however, the data were insufficient to determine the optimal regimen.

## 1. Introduction

Lymphoma is a common hematopoietic tumor in dogs, with an estimated annual incidence of 13–114 per 100,000 dogs [1,2,3]. Some forms of canine lymphoma are classified anatomically (e.g., alimentary/gastrointestinal, cutaneous and subcutaneous, and multicentric/generalized) according to the site of the primary tumor site. Of these cancers, primary central nervous system lymphoma (PCNSL) rarely occurs in dogs [4,5]. The incidence of canine nervous system tumors is estimated to be 14.5 per 100,000 among the population at risk [6], and intracranial tumors are observed in 2.0% to 4.5% of dogs [7,8,9]. PCNSL and secondary central nervous system lymphoma (SCNSL) account for approximately 0.9–4.0% and 9.4–12% of all canine intracranial tumors, respectively [9,10,11].

The common treatment for canine lymphoma is multimodal chemotherapy. The effectiveness of primary and rescue protocols in dogs with non-CNS lymphoma has been extensively reported. However, because most anticancer drugs for lymphoma cannot penetrate the blood-brain barrier (BBB), a standard protocol for canine PCNSL and SCNSL has not been established. The prognosis of canine CNS lymphoma treated with medical treatment has been reported to be poor (the survival times of dogs treated with chemotherapy alone or prednisolone alone were 42–171 days or 5–187 days, respectively [4,12]). Nimustine (ACNU) belongs to the same class of drugs, nitrosourea alkylating agents, as lomustine (CCNU). Nimustine is primarily used in the treatment of human intracranial neoplasias, such as gliomas, because of its high permeability across the BBB [13,14,15]. To date, the efficacy of nimustine in the treatment of canine lymphoma has not been extensively reported [16].

We report two cases of canine PCNSL or SCNSL that were treated with combined chemotherapy of nimustine and prednisolone, wherein the dogs achieved complete or nearly complete remission of neurological symptoms and long-term survival with some problematic but not life-threatening adverse effects. Adverse effects were evaluated according to the Veterinary Cooperative Oncology Group–Common Terminology Criteria for Adverse Events v2 (VCOG-CTCAE v2) [17]. The neutrophil counts in blood were rarely evaluated in this study; therefore, for the purpose of grading neutropenia, we assumed that 90 percent of the white blood cell (WBC) count was the neutrophil count.

## 2. Case Presentation

### 2.1. Case 1

A 3-year-old intact female toy poodle, weighing 2.2 kg, was referred to the Miyazaki University Veterinary Teaching Hospital (MUVTH) (on the first day of illness) because of acute pain and paralysis of the four limbs of unknown origin; additionally, the patient experienced an episode of medial subluxation of the right patella. No alimentary or respiratory symptoms were observed.

**Figure 1:**
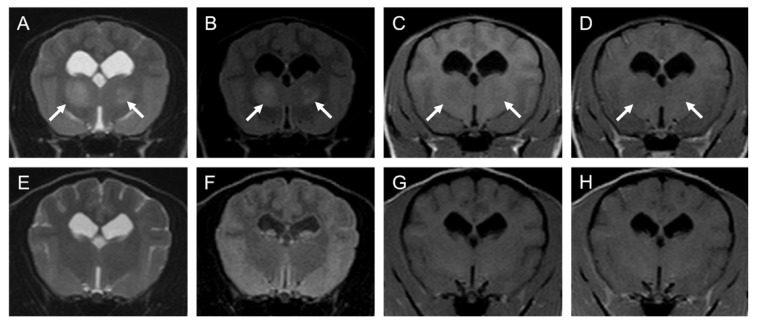
MRI images of suspected PCNSL in thalamus of a 3-year-old female toy poodle (Case1): (**A**–**D**); 5th day (before chemotherapy), (**E**–**H**); 233th day (after fourth injection of nimustine). Transverse T2-WI (**A**,**E**), FLAIR (**B**,**F**), T1-WI (**C**,**G**), T1-WI post-contrast (**D**,**H**). The left side of the patient is on the right side of the image. Arrows point to the suspected neoplastic lesion. Note that these lesions disappeared at the end of treatment. FLAIR: fluid-attenuated inversion recovery, T1-WI: T1-weighted image, T2-WI: T2-weighted image.

**Figure 2:**
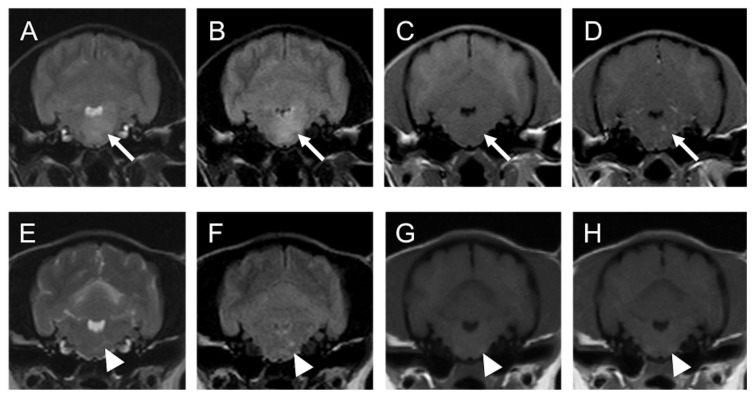
MRI images of suspected PCNSL in medulla oblongata of a 3-year-old female toy poodle (Case1): (**A**–**D**); 5th day (before chemotherapy), (**E**–**H**); 233th day (after fourth injection of nimustine). Transverse T2-WI (**A**,**E**), FLAIR (**B**,**F**), T1-WI (**C**,**G**), T1-WI post-contrast (**D**,**H**). The left side of the patient is on the right side of the image. Arrows point to the suspected neoplastic lesion. Note that these lesions are faintly enhanced after injection of contrast medium (gadolinium) on T1WI and disappeared at the end of treatment. Arrowheads point to the suspected non-neoplastic lesion. Note that these lesions are not enhanced after injection of contrast medium (gadolinium) on T1WI. FLAIR: fluid-attenuated inversion recovery, T1-WI: T1-weighted image, T2-WI: T2-weighted image.

A general physical examination, including palpation and auscultation, revealed no swelling of the superficial lymph nodes. Neurological examination revealed spontaneous horizontal nystagmus and disturbed proprioception in both forelimbs. Blood tests yielded no abnormal findings, and no atypical lymphocytes were observed. Plain radiographs of the thorax and abdomen also revealed no abnormalities. Magnetic resonance imaging (MRI) of the brain and cervical spine with the 0.4-T Aperto Inspire^®^ system (Hitachi Medical Co., Tokyo, Japan) was performed at Kagoshima University Veterinary Teaching Hospital (KUVTH) four days after the initial presentation. Lesions with high signal intensity were observed in both the thalamus and the left medulla oblongata on T2-weighted (T2W) and fluid-attenuated inversion recovery (FLAIR) images. On T1-weighted (T1W) images, these lesions were faintly enhanced by 0.2 mL/kg of gadolinium (ProHance^®^; Eisai, Tokyo, Japan), injected intravenously (Figure 1 and Figure 2).

Cerebrospinal fluid (CSF) examination revealed an elevated nucleated cell count (230 cells/μL), consisting mainly of typical lymphocyte-like and small- or medium-sized, immature lymphocyte-like cells. CSF clonality analysis was performed by a commercial laboratory (Canine-lab Co., Ltd.; Tokyo, Japan) using PCR amplification of immunoglobulin and T-cell receptor (Ig/TCR) genes: PCR for antigen receptor rearrangements (PARR). The analysis revealed B-cell monoclonal proliferation. All these results indicated a diagnosis of PCNSL or possibly SCNSL.

On the 21st day of illness, combined chemotherapy with nimustine and prednisolone was initiated. The first intravenous dose of nimustine (NIDRAN^®^; Daiichi Sankyo, Inc., Tokyo, Japan) was established at 50 mg/m^2^ in accordance with previous findings in humans [18] and a dog (K. Fujiwara, 2007; publication of clinical hematology information in a Japanese commercial magazine). Thereafter, nimustine was infused three more times; each dose was reduced by 10% (5 mg/m^2^) considering the decrease in WBC and blood lymphocyte counts. The interval of infusion was also adjusted as needed (28–62 days) until either the WBC count, lymphocyte count, or both recovered. Metoclopramide (0.5 mg/kg) and famotidine (0.4 mg/kg) were administered intravenously immediately before each nimustine infusion. Prednisolone was initiated at 2 mg/kg once per day and tapered to 0.5 mg/kg every other day. Although some adverse effects occurred, such as inappetence, leukocytopenia, thrombocytopenia, and elevation of serum alanine (ALT) and aspartate (AST) aminotransferase levels, all were temporary and not life-threatening (Table 1).

On the 233rd day of illness (80 days after the fourth nimustine infusion), an MRI was performed again at KUVTH. On the MRI, the lesions observed previously had mostly disappeared except for slightly high signal intensity on T2W and FLAIR images and no contrast enhancement by gadolinium on T1W images (Figure 1 and Figure 2). The faint lesions were considered to represent gliosis or the loss of neurons. Long-term follow-up was conducted mainly through telephone interviews with the owner. The dog lived for more than 7 years (2583 days) after the initial presentation without any neurological symptoms.

### 2.2. Case 2

A 5-year-old spayed female toy poodle, weighing 4.8 kg, was referred to MUVTH (on the 1st day of illness) because of a two-month history of repeated generalized seizures and circling to the left. No alimentary or respiratory symptoms were observed.

**Figure 3:**
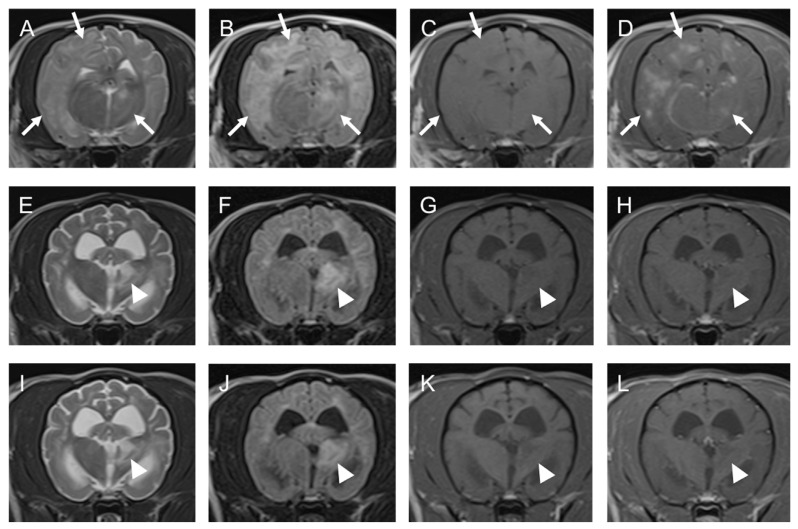
MRI images of suspected PCNSL in cerebral white matter and thalamus of a 5-year-old spayed female toy poodle (Case 2): (**A**–**D**); 1st day (before chemotherapy), (**E**–**H**); 176th day (after fourth injection of nimustine), (**I**–**L**); 345th day Transverse T2-WI (**A**,**E**,**I**), FLAIR (**B**,**F**,**J**), T1-WI (**C**,**G**,**K**), T1-WI post-contrast (**D**,**H**,**L**). The left side of the patient is on the right side of the image. Arrows point to the suspected neoplastic lesion. Note that these lesions are heterogeneously enhanced after injection of contrast medium (gadolinium) on T1WI and disappeared at the end of treatment. Arrowheads point to the suspected non-neoplastic lesion. Note that these lesions are not enhanced after injection of contrast medium (gadolinium) on T1WI and not changed between the 176th day and 345th day. FLAIR: fluid-attenuated inversion recovery, T1-WI: T1-weighted image, T2-WI: T2-weighted image.

A general physical examination, including palpation and auscultation, revealed no swelling of superficial lymph nodes. Neurological examination revealed circling to the left, decreased postural responses in four limbs, and hypermetria in both forelimbs. Blood tests yielded no abnormal findings except a mild elevation of serum ALT and AST levels. This was suspected to be an adverse effect of the temporary administration of prednisolone by the referring veterinarian. No atypical lymphocytes were evident on blood film examination. Plain radiographs of the thorax and abdomen also revealed no abnormalities. An MRI (with 3.0-T Vantage Titan 3T^®^; Canon Medical Systems, Otawara, Tochigi, Japan) of the brain and cervical spine was performed in MUVTH on the day of presentation, and diffuse lesions of high signal intensity, primarily in the right cerebral white matter, the left thalamus, and the cerebellum, were observed on the T2W and FLAIR images. On the T1W images, these lesions were mildly enhanced by 0.2 mL/kg of gadolinium (Magnevist^®^, now discontinued; Bayer Yakuhin Ltd., Osaka, Japan), injected intravenously (Figure 3).

**Figure 4:**
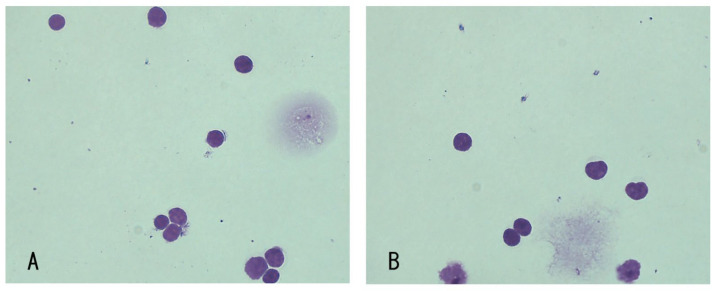
Histological findings of cerebrospinal fluid (CSF) cytology for Case 2. (CSF smear, Diff- Quik stain, 400×) CSF was collected just after the first MRI acquisition (**A**,**B**).

The CSF examination revealed pleocytosis (990 cells/μL); the cells consisted mainly of typical lymphocyte-like cells and small- or medium-sized, immature lymphocyte-like cells (Figure 4).

Prednisolone and cyclosporine were administered for suspected nonpurulent encephalitis until the results of the CSF clonality analysis were available. A CSF clonality analysis was performed, as in Case 1, using Ig/TCR gene PCR, which showed B-cell monoclonal proliferation. All these results indicated a diagnosis of PCNSL, or possibly SCNSL.

On the 14th day of illness, combined chemotherapy with nimustine and prednisolone was initiated in accordance with the diagnosis of lymphoma, and cyclosporine was discontinued, although clinical features had already improved since commencing treatment. The dose of nimustine to be infused was established at 30–35 mg/m^2^ based on our previous experience with Case 1. Nimustine was infused intravenously three more times. The doses and the interval between infusions (30–39 days) were adjusted based on the decreasing WBC and lymphocyte counts in the blood. Metoclopramide (0.5 mg/kg) and famotidine (0.4 mg/kg) were administered intravenously immediately before each nimustine infusion. Prednisolone was started at 2 mg/kg once per day and tapered to 0.25 mg/kg every other day. No generalized seizure was observed after the first nimustine infusion or throughout the protocol. On the second nimustine infusion, the decreased postural responses of four limbs and hypermetria of both forelimbs were ameliorated. However, circling to the left continued (in fact, for the rest of the dog’s life). Although all of these were temporary, some adverse effects, such as leukocytopenia (grades 1–3 of neutropenia) and elevation of the serum alkaline phosphatase level, were noted (Table 1). On the 176th day of illness (58 days after the fourth nimustine infusion), an MRI was repeated at MUVTH. On the MRI, the lesions previously observed had mostly disappeared except for slightly high signal intensity in the cerebral white matter and left thalamus on the T2W and FLAIR images. No enhancement by gadolinium appeared on the T1W images (Figure 3). The faint lesions were considered to represent gliosis or the loss of neurons. A CSF examination revealed no abnormalities. On the 345th day of illness (227 days after the fourth nimustine infusion), a third MRI was performed at MUVTH at the owner’s request, and the findings were comparable with those of the second MRI examination (Figure 3). A CSF examination revealed no abnormalities, and a lymphocyte clonality analysis yielded negative results. Beginning on the 427th day of the illness, intermittent seizures were observed. They were thought to be caused by brain damage due to the lymphoma, and antiepileptic treatment with zonisamide and potassium bromide was initiated. In addition, on the 1003rd day of illness, transitional cell carcinoma (TCC) of the bladder was diagnosed, followed by end-stage renal disease, and the dog died on the 1218th day of illness. It reduced the mean monthly frequency of seizures to 0.58 during the year before the dog died. TCC was confirmed via histopathological examination. Other than persistent circling to the left and intermittent seizures, the dog did not exhibit any relapse of neurological deficits until the 1107th day of illness. However, mild paresis of both hindlimbs started after the resection of the bladder tumor. Because no other neurological deficits appeared, the cause of the paresis was not suspected to be a relapse of the intracranial lymphoma. However, no detailed examination has been performed to verify the cause. During the treatment, the referring veterinarian temporarily administered prednisolone for Case 2.

## 3. Discussion

For suspected PCNSL, two dogs were treated with nimustine, which enabled long-term survival without relapse for more than 1000 days. Although chemotherapy with nimustine for PCNSL and SCNSL has been reported in humans, we found no previous reports of such treatment in affected dogs. The median length of survival in canine cases of PCNSL, including brain and spinal lymphoma, after combination therapy with surgery, radiation, and chemotherapy has been reported to be 254 days (range, 91–1939 days; *n* = 3). According to the same report, the survival time after combination therapy with surgery and radiation therapy was 40 and 379 days, the survival time after combination therapy with surgery and chemotherapy was 261 days, the survival time after combination therapy with radiation and chemotherapy was 268 and 179 days, the median length of survival after chemotherapy alone was 86 days (range 42–171 days; *n* = 4), the survival time treated with prednisolone alone was 187 days, and the survival time without treatment was 1 day. (These data were extracted from the research by Larue et al. [4] and recalculated.) Furthermore, one dog with PCNSL treated with prednisolone alone survived for 5 days [12]. The two dogs that we treated with nimustine survived far longer than those in previous reports. Kodama et al. reported the long-term survival of a cat with large granular lymphocyte (LGL) lymphoma by nimustine treatment [19]. LGL lymphoma in cats is a rare subtype and is derived from cytotoxic T cells or natural killer cells [20,21]. Our two cases were diagnosed with canine B-cell lymphoma. Further consideration is needed about the efficacy of nimustine for lymphoma of other species or non-B-cell lymphoma.

Lomustine, which, like nimustine, is a nitrsourea alkylating agent, has high permeability across the BBB and has been used to treat brain tumors in dogs [22,23]. Its use has also been reported in the rescue and primary protocols of chemotherapy for non-central nervous system lymphoma in dogs [24,25]. Both nimustine and lomustine need to be administered only once every 3–4 weeks, so the treatment protocols are simple and convenient. However, lomustine is an oral drug; in cases of alimentary problems such as anorexia or inappetence, detailed dose adjustment and administration are difficult. Furthermore, lomustine has not been approved or sold in Japan; therefore, its use has been limited there. In contrast, nimustine is an injection drug that has been approved and is available in Japan; thus, its use has not been limited. Furthermore, in cases of alimentary problems such as anorexia or inappetence, detailed adjustment of the dosage and administration is easy.

The most commonly reported adverse effect of nimustine is delayed myelosuppression in humans; thus, the complete blood cell (CBC) count must be monitored during treatment. A previous study showed that a decrease in neutrophil counts was observed in dogs treated with nimustine, with nadir occurring 7 days after administration, and decreased thrombocyte counts were also observed in the dogs, with nadir occurring between 7 and 21 days after administration of nimustine [26]. Therefore, we checked the count of CBC and thrombocytes 7–10 days after each administration of nimustine and just before the next administration. In Case 1, moderate leukocytopenia was observed one week after each of the first three nimustine infusions, and grade 3 thrombocytopenia was observed one week after the third and fourth nimustine infusions, with a dosage of 50–35 mg/m^2^ of body surface. This adverse effect was temporary, and the total WBC and thrombocyte counts recovered without the need for specific clinical interventions. However, the recovery was delayed as the dose and number of infusions increased. We, therefore, established a nimustine dose of 30–35 mg/m^2^ for Case 2. Even at this dose, moderate-to-severe leukocytopenia (grade 3 neutropenia) was observed. In this study, we assumed that 90% of the total WBC count was the neutrophil count after nimustine administration for the purpose of grading neutropenia because the follow-up blood examination just before the next nimustine administration did not reveal any abnormalities in the subpopulation of WBC or total WBC count. However, in clinical use, this grading method is not inappropriate or risky, especially in lymphoma with extensive peripheral blood or reactive lymphocytosis and monocytosis. It may lead to a misunderstanding of the subpopulation of WBC and an overlooking of critical findings. To the best of our knowledge, hepatotoxicity associated with nimustine has not been reported in dogs, although lomustine has been reported to have cumulative and irreversible hepatotoxicity [27,28]. On the other hand, cumulative hepatotoxicity has been reported in cats following multiple nimustine infusions [29]. However, the hepatotoxicity was considered to be only mild in that report. Although the elevation of serum ALP, ALT, and AST levels was also documented in our study, it was again not severe (grade 1–2), and treatment with a liver protectant was not considered necessary. Therefore, nimustine might be less toxic than lomustine; however, further studies are mandatory to determine the appropriate protocol to follow when administering nimustine to dogs. Moreover, nimustine has been reported to be a vesicant anticancer drug according to some literature [30]; therefore, clinicians must ensure that nimustine does not leak out of the blood vessels when injected intravenously.

In accordance with the treatment results and the adverse events that followed in our cases, we determined that the dose of nimustine for canine PCNSL or SCNSL with intravenous infusion was 25–30 mg/m^2^ every 3–4 weeks, a total of four times; however, the data were insufficient to determine the optimal regimen. Chemotherapies with nimustine were initiated in Cases 1 and 2 in 2010 and 2016, respectively. In 2014, Takahashi et al. reported that the dose of nimustine for tumor-bearing dogs was 25 mg/m^2^ every 3 weeks in a phase I trial [26]. Therefore, in Case 1, we administered nimustine at the maximum dose of 50 mg/m^2^, mainly based on some reports in humans [31,32]. This dose was apparently high in dogs. Therefore, it is desirable to perform an appropriate phase II trial in order to establish an optimal regimen.

In our two cases, B-cell lymphomas were diagnosed through CSF clonality analysis. According to the revised WHO classification, almost all human PCNSL is classified as diffuse large B-cell lymphoma (DLBCL) [33]. In addition, it was also reported that canine B-cell lymphomas were almost classified as DLBCL [34]. According to the histological findings of the CSF cytology in our cases, most of the cells observed were lymphocyte-like cells with medium to large nuclei and no granules. It has some similarities to DLBCL; however, we could not classify the lymphomas in this study. Ehrhart et al. reported that the PARR assay showed satisfactory performance in the discrimination of B-cell lymphoma from non-lymphoma and T-cell lymphoma, with an accuracy of 95–100% [35]. However, a strong inflammatory reaction and cross-lineage rearrangement may potentially lead to false-positive monoclonal results and a misdiagnosis of clonality [36,37]. One of the strong limitations of this study is the lack of histopathological examination for diagnosis.

Lesions of high signal intensity were observed in the thalamus and the medulla oblongata in Case 1 and in the cerebral white matter, the thalamus, and the cerebellum in Case 2 on the T2W and FLAIR images. In feline CNS and nasal lymphoma, the usefulness of the apparent diffusion coefficient (ADC) value has been reported for the diagnosis of lymphoma [38,39], as described for human CNS lymphoma [40]. In our cases, diffusion-weighted imaging was not performed because the MRI equipment was a low-field device in Case 1, and a low ADC value was not clearly observed in Case 2. In the latter, the lesions were diffuse throughout the whole cerebrum and cerebellum with the presence of peritumoral edema; therefore, the change in ADC value might not be detected. After the completion of the full treatment protocol, only subtle or faint lesions remained visible on MRI and were suspected to be associated with gliosis, loss of neurons, or both. In support of this hypothesis, these lesions were not enhanced by gadolinium on the T1W images after treatment. Moreover, in Case 2, the remaining high-intensity lesions on the T2W and FLAIR images did not change on the later MRI after 169 days of nontreatment, except for the temporary use of prednisolone by the referring veterinarian. The confirmed circling to the left and intermittent seizures observed in Case 2 were also suspected to be associated with these non-neoplastic lesions. According to the guidelines on the diagnosis and management of PCNCL in humans [41], the disappearance of gadolinium-enhanced lesions on the MRI is a criterion for the response to be considered a complete response. Our two cases met this requirement; therefore, we hypothesized that the chemotherapy with nimustine could achieve a complete clinical response. Because the start of chemotherapy was delayed in Case 2, the central nervous system probably experienced irreversible damage. This outcome indicates that lymphoma should be diagnosed and treated as early as possible.

## 4. Conclusions

To the best of our knowledge, this is the first report of successful long-term survival following primary chemotherapy with nimustine for canine PCNSL or SCNSL. In our two cases, B-cell lymphomas were diagnosed through CSF clonality analysis; however, some PCNSL and SCNSL have been reported to be T-cell lymphomas in dogs [12,34]. Our findings indicate the effectiveness of nimustine as a primary chemotherapy for PCNSL or SCNSL in dogs. Further investigation is needed to establish an optimal protocol for B-cell lymphomas and verify the effectiveness of nimustine against T-cell lymphoma.

## Figures and Tables

**Table 1:** Count of total white blood cell (WBC), neutrophil, lymphocyte, thrombocyte, and chemistry tests before and after each nimustine injection.

		1st Injection		2nd Injection		3rd Injection		4th Injection	
		0 Day	After	0 Day	After	0 Day	After	0 Day	After
Case 1	Blood examination day after injection (day)		7		7		8		9
	WBC (cells/μL)	17,100	1300 ^††^	10,700	1500 ^††^	8700	1200 ^††^	11,100	4300
	neutrophil (cells/μL)	N.D.	N.D.	8453	N.D.	7134	N.D.	7992	N.D.
	Lymphocyte (cells/μL)	N.D.	N.D.	1445	N.D.	304	N.D.	1221	N.D.
	Thrombocyte (cells/μL)	591,000	116,000	280,000	130,000	134,000	48,000 ^†††^	119,000	27,000 ^†††^
	Alkaline phosphatase (U/L)	427 ^††^ (1.68 × ULN)	N.D.	311 ^†^ (1.22 × ULN)	N.D.	N.D.	N.D.	N.D.	N.D.
	Alanine aminotrasferase (U/L)	218 ^††^ (2.79 × ULN)	N.D.	62 (<ULN)	N.D.	42 (<ULN)	N.D.	N.D.	N.D.
	Aspartate aminotrasferase (U/L)	33 (<ULN)	N.D.	27 (<ULN)	N.D.	N.D.	N.D.	N.D.	N.D.
	Dose of nimustine (mg/m^2^)	50		45		40		35	
Case 2	Blood examination day after injection (day)		7		6		7		7
	WBC (cells/μL)	21,000	6000	22,000	800 ^†††^	18,900	700 ^†††^	16,500	1600 ^†^
	neutrophil (cells/μL)	N.D.	N.D.	21,560	N.D.	17,199	N.D.	13,695	N.D.
	Lymphocyte (cells/μL)	N.D.	N.D.	220	N.D.	593	N.D.	495	N.D.
	Thrombocyte (cells/μL)	281,000	201,000	294,000	298,000	275,000	274,400	480,000	119,000
	Alkaline phosphatase (U/L)	1149 ^††^ (4.52 × ULN)	N.D.	895 ^††^ (3.52 × ULN)	N.D.	597 ^†^ (2.35 × ULN)	N.D.	873 ^††^ (3.44 × ULN)	N.D.
	Alanine aminotrasferase (U/L)	266 ^††^ (3.41 × ULN)	N.D.	119 ^††^ (1.53 × ULN)	N.D.	79 ^†^ (1.01 × ULN)	N.D.	93 ^†^ (1.19 × ULN)	N.D.
	Aspartate aminotrasferase (U/L)	45 ^†^ (1.02 × ULN)	N.D.	26 (<ULN)	N.D.	21 (<ULN)	N.D.	25 (<ULN)	N.D.
	Dose of nimustine (mg/m^2^)	35		35		32		30	

0 day: the values just before nimusutine injection on the day; N.D.: data is not available or not examined; ULN: upper limit of normal (from reference range measured with an animal dry-chemistry multianalyzer, “Fuji-DRI-CHEM 7000 V”; Fujifilm, Tokyo, Japan); ^†^: grade 1 adverse event according to the VCOG-CTCAE v2; ^††^: grade 2 adverse event according to the VCOG-CTCAE v2; ^†††^: grade 3 adverse event according to the VCOG-CTCAE v2; Note: the total WBC count was graded instead of neutrophil subpopulation (as mentioned in the text).

## Data Availability

The data presented in this study are available in the manuscript.
